# Esoteric Diagnostic Considerations for Small Round Cell Tumors in Biopsy Specimens With Extensive Negative Immunohistochemical Profiles: Utilizing Subtle Histopathological Features Prior to Molecular Testing

**DOI:** 10.1155/crom/5186729

**Published:** 2025-08-04

**Authors:** Dong Ren, Ryan O'connell

**Affiliations:** Departments of Pathology and Laboratory Medicine, University of California Irvine Medical Center, Orange, California, USA

**Keywords:** diagnostic consideration in biopsy, extensive immunohistochemical workup with negative results, small round cell tumor

## Abstract

Small round cell tumors (SRCTs) are characterized by primitive round cells and a broad differential diagnosis due to their undifferentiated nature, making their diagnosis particularly challenging. Molecular testing is often essential for definitive classification; however, subtle histomorphological features can significantly narrow the differential diagnosis. Here, we present the case of a 44-year-old male who presented with a painless mass (up to 15.6 cm) in the left thigh. Histologic examination of the biopsy revealed solid sheets of monotonous small round cells with scant cytoplasm, hyperchromatic nuclei, and conspicuous nucleoli within the edematous to myxoid stroma. Notably, capillary-sized blood vessels were present throughout the tumor, which made BCOR-rearranged sarcomas, myxoid liposarcoma with small cell morphology, and GLI1-altered soft tissue tumors the main differential diagnoses. Classic morphology of myxoid liposarcoma was not present. Immunohistochemical (IHC) staining revealed that the tumor cells were diffusely positive for SOX11 but negative for SATB2, CD56, S100, and TLE1. This immunophenotype, combined with the histological findings, strongly suggested a diagnosis of myxoid liposarcoma with high-grade features. Fluorescence in situ hybridization (FISH) analysis confirmed a DDIT3 rearrangement, supporting this diagnosis. We hope this case will enhance pathologists' understanding and recognition of the importance of utilizing subtle histologic features to establish the differential diagnosis and accurately diagnose SRCTs in biopsy specimens prior to molecular testing.

Small round cell tumors (SRCTs) refer to a group of neoplasms characterized by round primitive cells. Ewing's sarcoma, rhabdomyosarcoma, and lymphoma are the most commonly encountered SRCTs in clinical practice, regardless of the patient's age or tumor location [1]. Additionally, location-related SRCTs, such as retinoblastoma, neuroblastoma, hepatoblastoma, and nephroblastoma, are also frequently observed [1]. Other diagnostic considerations include poorly differentiated carcinoma and melanoma. Diagnosing SRCTs can be challenging due to the undifferentiated nature of the tumor and the broad range of differential diagnoses. The histologic growth pattern in resection specimens often provides key clues for pathologists. However, this may be absent in biopsy specimens, making the diagnosis of SRCTs particularly difficult.

Here, we present the case of a 44-year-old male who presented with a painless mass in the left thigh for approximately a year that increased significantly in size, from 8.9 to 15.6 cm. MRI of the lesion showed a heterogeneous solid mass without myxoid component identified. Histologic examination of the biopsy revealed solid sheets of monotonous small round cells with scant cytoplasm, hyperchromatic nuclei, and conspicuous nucleoli, embedded within edematous to myxoid stroma (Figures 1A, 1B, and 1C). Notably, capillary-sized blood vessels were present throughout the tumor (Figure 1A,B). Nuclear pleomorphism and necrosis were not identified. Extensive immunohistochemical (IHC) staining revealed that the tumor cells were negative for pan-keratin, EMA, CD45, CD99, desmin, SMA, MSA, H-caldesmon, myogenin, MyoD1, CD31, ERG, and melanocytic markers, which ruled out several commonly encountered SRCTs, including poorly differentiated carcinoma, lymphoma, Ewing's sarcoma, rhabdomyosarcoma, leiomyosarcoma, angiosarcoma, and melanoma. Due to the limited nature of the biopsy specimen, other mesenchymal neoplasms with round cell morphology were considered, including but not limited to the following:

• BCOR-rearranged sarcomas (characterized by uniform primitive small round to ovoid cells arranged in solid sheets or nests, capillary network, and myxoid stroma; positive for CD99, SATB2, and cyclin D1)
• Myxoid liposarcoma with small cell morphology (nodules and solid sheets of round cells, capillary vessels, and myxoid stroma positive for SOX11) [2]
• Extraskeletal myxoid chondrosarcoma with high-grade features (large, epithelioid round cells; variable expression of S100, CD117, synaptophysin, INSM1)
• Malignant glomus tumor with round cell morphology (solid nests of small round cells with high-grade nuclei, positive for SMA, pericellular type IV collagen)
• Extraskeletal mesenchymal chondrosarcoma (uniform small round cells in myxoid to chondroid stroma, positive for SOX9, CD99, and ERG) [3]
• Small cell variant of extraskeletal osteosarcoma (uniform small round cells with lace-like osteoid, positive for SATB2)
• GLI1-altered soft tissue tumors (monomorphic round to epithelioid cells with multinodular growth patterns, a rich capillary network, and myxoid to collagenous stroma; diffusely positive for CD56, variable expression of S100) [4]
• ALK-rearranged epithelioid mesenchymal neoplasm (sheets, cords, or small clusters of round epithelioid cells in myxohyaline stroma; diffusely positive for ALK1, variable positivity for CD34, S100, EMA, and SMA) [5]
• Dedifferentiated solitary fibrous tumor (SFT) (sheets and nests of poorly differentiated round cells, hemangiopericytoma-like vessels, positive for CD34, STAT6)
• Poorly differentiated synovial sarcoma (solid nests of poorly differentiated round cells, positive for TLE1 and SS18)
• NUT carcinoma/sarcoma (sheets of primitive, monotonous round cells with squamous differentiation and intratumoral acute inflammation, positive for cytokeratin, CD99, NUT1, p63, and p40)
• Myoepithelial carcinoma with undifferentiated round cell morphology (sheets of monotonous round cells, positive for keratin and S100)

Given the presence of capillary-sized blood vessels and myxoid stroma in the lesion, the primary differential diagnoses included BCOR-rearranged sarcomas, myxoid liposarcoma with small cell morphology, and GLI1-altered soft tissue tumors. Interestingly, strong SOX11 expression has been reported in all cases of myxoid/round cell liposarcoma (100%) [2], as well as in various other mesenchymal tumors, including rhabdomyosarcomas (90%), synovial sarcomas (36%), and malignant peripheral nerve sheath tumors (MPNSTs) (24%) [2]. Further IHC revealed that the tumor cells were diffusely positive for SOX11 (Figure 1D) but negative for SATB2, CD56, S100, and TLE1. This immunophenotype, combined with the histological findings, strongly suggested a diagnosis of myxoid liposarcoma with high-grade features. Fluorescence in situ hybridization (FISH) analysis confirmed a DDIT3 rearrangement, supporting this diagnosis.

As molecular technologies continue to advance, molecular analysis plays an increasingly critical role in the diagnosis and classification of SRCTs. Techniques such as reverse transcription-polymerase chain reaction (RT-PCR), FISH, next-generation sequencing (NGS), and optical genome mapping (OGM) have become essential tools in this context [6]. While histopathological and IHC evaluations are useful in narrowing the differential diagnosis, molecular diagnostics are often required to establish a definitive diagnosis—particularly in challenging or ambiguous cases, where SRCTs exhibit significant morphologic overlap. In the present case, although the histomorphological features and diffuse SOX11 positivity suggested a diagnosis of myxoid liposarcoma with high-grade features, molecular testing (specifically, FISH analysis) was necessary to confirm the diagnosis. However, it is important to note that a pathologic diagnosis should not rely solely on molecular findings. The EWSR1::FLI1 fusion is a well-established hallmark of Ewing sarcoma [7]. However, Folpe et al. have described a novel entity—superficial neurocristic EWSR1::FLI1 fusion tumor—characterized by circumscribed and multinodular tumor consisting of nests of bland, round cells admixed with hyalinized collagenous bands containing spindled cells [8, 9]. Therefore, molecular analysis, when interpreted in the appropriate histopathologic context, is most effective in rendering an accurate and definitive diagnosis.

This case offers a comprehensive diagnostic approach for SRCTs by utilizing subtle histopathological features in biopsy specimens before molecular testing, especially after excluding common SRCTs and when classic features of some tumors with round cell transformation may not be fully appreciated. We hope this case enhances pathologists' understanding of the differential diagnosis of SRCTs, particularly in limited biopsy samples.

## Figures and Tables

**Figure 1:**
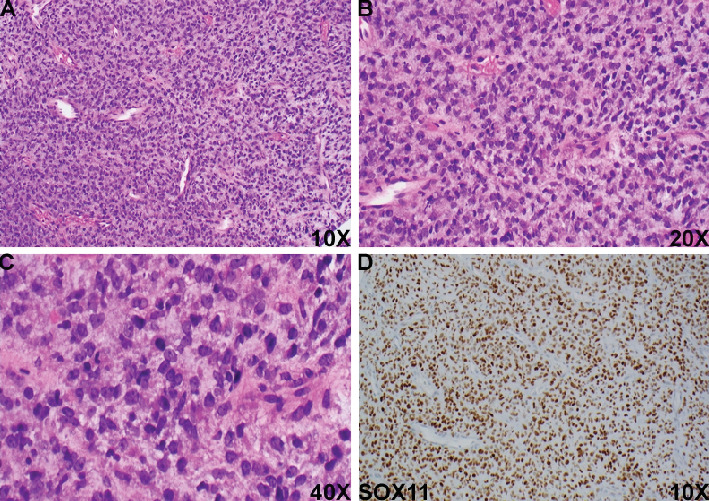
Histologic findings and SOX11 immunoreactivity of the tumor. (A–C) Representative histologic finding of the tumor specimen shows solid sheets of monotonous primitive small round cells with capillary-sized blood vessels embedded within edematous to myxoid stroma, (A) 10X, (B) 20X, and (C) 40X. (D) The tumor cells are strongly and diffusely positive for SOX11 (nuclear staining), 10X.

## Data Availability

The data that support the findings of this study are available on request from the corresponding authors. The data are not publicly available due to privacy or ethical restrictions.
